# Studies on the ABH-Iso-Agglutinins in serum, saliva and milk from mothers with “Bombay” (Oh) phenotype

**DOI:** 10.4103/0973-6247.44478

**Published:** 2009-01

**Authors:** S. R Joshi, K. Vasantha, Y. S. Iyer, S. Kulkarni, S. Jadhav

**Affiliations:** *Bombay Red Cross Blood Center, Mumbai, India*; 1 *National Institute of Immunohaematology, Mumbai, India*

**Keywords:** ABH-iso-antibodies, bombay phenotype, secretions

## Abstract

**Background:**

ABO blood group iso-antibodies are naturally occurring antibodies found in serum and other body fluids.

**Methods:**

Serum, saliva and milk samples from 5 mothers identified as “Bombay” phenotype were tested for ABH-iso-antibodies by routine serological techniques.

**Results:**

All the five mothers showed presence of iso-antibodies in the samples tested. Higher titer values in milk than their serum were observed on subjects whose samples were collected in immediate post-partum phase as compared to those whose samples were collected after a lapse of a few months.

**Conclusion:**

High titer iso-agglutinins against ABH antigens were detected in milk samples besides their presence in saliva as well as serum.

## Introduction

Iso-agglutinins to the ABH antigens are naturally occurring alloantibodies regularly found in plasma or serum of an individual lacking the corresponding antigen on red cells. The reciprocal relationship of these antibodies in serum to corresponding antigen on red cells helps in confirming the ABO blood group of a person and warrants the use of homologous blood in transfusion. Besides plasma, the ABO iso-antibodies have also been detected in other body fluids and secretions.[1–5] Badakere and Bhatia[6] found the low titer ABO agglutinins in saliva of Indians from Bombay. To the best of our knowledge, the antibodies to ABH antigens in milk/saliva from ‘Bombay’ phenotype have not been reported so far. The present report deals with such a study among mothers with the rare ‘Bombay’ phenotype.

## Materials and Methods

Blood (clotted), saliva and milk samples from recently delivered “Bombay” group women were collected in sterile containers and transported to the laboratory by maintaining an appropriate cold chain and tested for antibody reactivity on the same day of collection. The remainders of the serum, saliva and milk samples were kept frozen at −20°C for further studies. Native saliva, being hypotonic in nature, was diluted with equal volume of normal saline so as to obviate osmotic hemolysis in the test. Titer values were obtained by semi-quantitative method using serial dilution of fluid in normal saline. The red cells of appropriate ABO group were used in 2% concentration in normal saline. The test was incubated at room temperature for 30 minutes and results were read after centrifuging at 1000 rpm for 1 minute. For absorption studies, equal volumes of thrice washed packed red cells of appropriate ABO groups were mixed with the samples and incubated for 1 hour at room temperature. Absorbed samples were separated after hard centrifugation (3500 rpm for 5 minutes). Antibody elution was carried out by heat elution in 56°C water-bath by heating the sensitized red cells that were washed thrice with chilled saline and suspended in 50% concentration. The immunoglobulin nature of the iso-agglutinins in milk was determined by the column chromatography technique.[7] Titration scores were calculated as per standard procedure.[8]

## Results

The samples of the five Bombay phenotype mothers showed presence of anti-A, anti-B and anti-H in saliva and milk besides being present in serum. Anti-A and anti-B iso-agglutinin strength ranged between 1:32 and 1:512 in serum and between 1:16 and 1:512 in milk but there was no appreciable difference for anti-A, anti-B and anti-H in saliva (range between 1:2 and 1:8). The findings are summarized in Table 1.

**Table 1:** ABH iso-antibody strength against group A1, B and O red cells in serum, milk and saliva from the mothers with “Bombay” phenotype

Mothers Gr Oh	Test Day#	Anti-A			Anti-B			Anti-H		
		
		Serum	Milk	Saliva	Serum	Milk	Saliva	Serum	Milk	Saliva
Sho	3	64	128	2	128	256	2	128	512	4
Ros	3	64	512	2	64	512	2	16	512	2
Lal	50	32	32	4	32	32	4	32	32	4
Kam	90	512	32	8	512	32	8	512	32	8
Apa	90	256	16	4	256	16	4	64	32	4

# day after post partum

**Figure 1:**
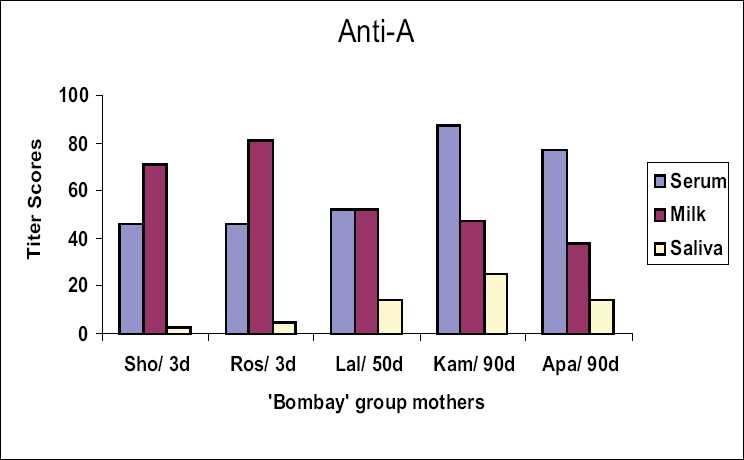
Titer scores for anti-A in serum, milk and saliva samples from the mothers with ‘Bombay’ phenotype, #d = sample collection-day after post partum

**Figure 2:**
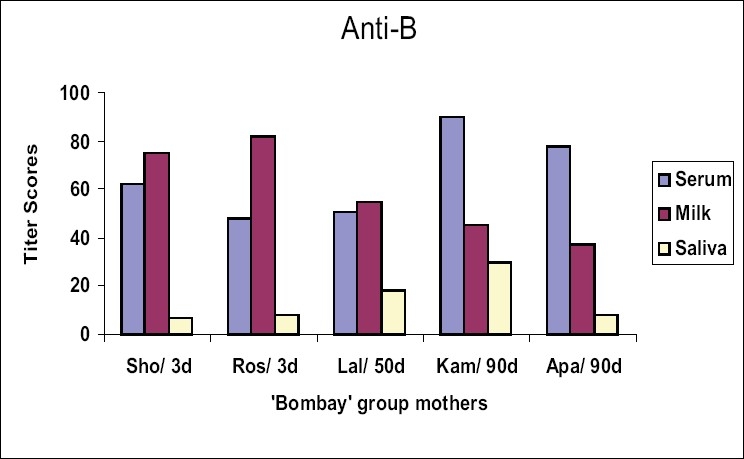
Titer scores for anti-B in serum, milk and saliva samples from the mothers with ‘Bombay’ phenotype, #d = sample collection-day after post partum

**Figure 3:**
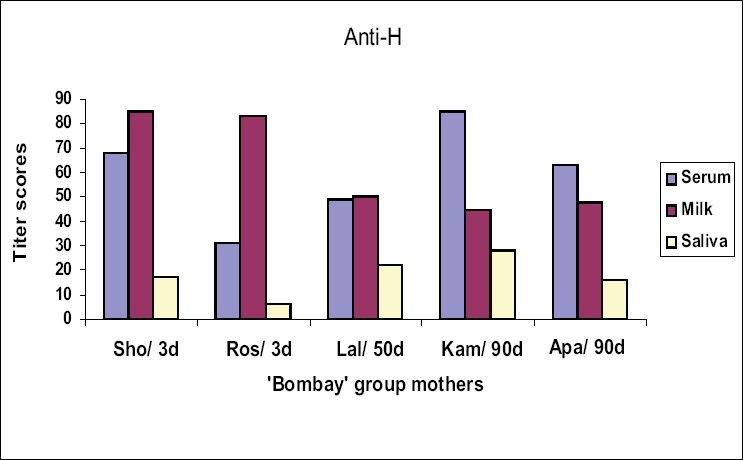
Titer scores for anti-H in serum, milk and saliva samples from the mothers with ‘Bombay’ phenotype, #d = sample collection-day after post partum

Iso-antibodies were found to be in higher concentration in milk than in serum samples of the mothers Sho and Ros who were tested within three days postpartum. There was no difference in levels of iso-antibody in Lal, who was tested after 50 days of delivery. Interestingly, the mothers Kam and Apa, whose samples were collected 90 days after delivery, showed a remarkable shift for higher titer values in serum as compared to milk. Salivary iso-agglutinins, though uniformly detected as low titer antibodies in all the 5 mothers tested, did not show such a shift in their antibody strength [Figure 1–3].

Absorption-elution studies were carried out on the milk sample of Sho. A single absorption with group A_1_ red cells removed reactivity for group A_1_, B and O red cells. On the other hand, absorption with group B cells removed reactivity for group B and group O red cells, leaving behind a low titer (1:2) anti-A_1_. Similarly, absorption with group O red cells removed reactivity for B and O red cells keeping a low titer (1:4 reactivity) for group A_1_ red cells. The findings are summarized in Table 2. Interestingly, eluate prepared by heat-elution method did not show reactivity for any of the red cells used in the absorption studies (Results are not shown).

The iso-antibodies in milk samples were found to be immunoglobulin A (IgA) in nature in all the 5 samples tested (Results are not shown).

**Table 2:** Showing reactivity in milk samples from Sho. after absorption with A, B and O cells

Test cells	Unabsorbed	Titer values in milk samples After absorption with		
		
		A cells	B cells	O cells
A	128	0	1:2	1:4
B	256	0	0	0
O	512	0	0	0

## Discussion

ABO iso-antibodies are not only confined to serum but are also present in various secretions including saliva,[2] cervical secretion,[3] tears,[4] and milk.[5] Salivary antibodies were more frequently found in group O individuals than in people with other blood groups.[2] Shlesiger[9] observed ABO-iso antibodies to be less detectable during pregnancy. In the present studies, all the mothers with ‘Bombay’ phenotype showed low-titer anti-A, anti-B and anti-H, which may be related to their pregnancies in the recent past.

An available study report in milk suggests that anti-A and anti-B have a higher titer in colostrums than in plasma from the same person.[5] The present study showed similar findings with interesting features for the levels of antibodies in serum vs. milk. A higher concentration of antibodies in milk than in serum was noticed among the mothers whose samples were collected within 3 days postpartum, whereas in a mother whose samples were collected 50 days after delivery showed the same strength of antibodies in milk as well as in serum. There was a dramatic shift in levels of the antibodies in milk and in serum of the other two mothers, whose samples were collected 90 days after delivery. In these mothers, their serum showed a higher titer value of antibodies as compared to their milk. Salivary iso-agglutinins, though uniformly detected as low titer antibodies in all the 5 mothers, did not show such a shift in their strength. It is likely that a higher concentration of these antibodies at delivery may have some bearing with its importance to provide passive immunity to the newborns to protect from certain infections.[10] IgA immunoglobulin specificity of the iso-antibodies in milk as observed in the present study and reported in literature reflects similarity to the secretory antibodies in milk.[5]

These observations are based on a very limited size of sample from a rare entity, and inferences made are only speculative in nature. Further study should involve a large sample size, not necessarily on the ‘Bombay’ phenotype but on other common ABO groups with iso-antibodies. Follow-up studies on the individual cases over a period of time may show a better picture on shift in level of iso-antibodies in serum and milk.

A single absorption had efficiently absorbed out iso-agglutinins from milk, though interestingly, eluate (heat) preparation did not show a strong reactivity. It is likely that the milk antibody may have a greater affinity to antigen giving a firm binding that becomes difficult to dissociate by the procedure of elution applied. Alternatively, antigen shed by heating might have neutralized the eluted antibody molecules depriving them of the reactivity from indicator red cells.

## References

[1] Race RR, Sanger R (1975). The ABO blood groups. Blood Groups in Man.

[2] Prokop O (1961). Studies on the secretion antibodies in saliva. Cong. Leg. Med., Vienna.

[3] Gershowitz H, Behrman SJ, Neel JV (1958). Hemagglutinins in uterine secretions. Science.

[4] Prokop O, Bundschuh G, Geserick G (1963). Uber blutgruppenreaktionen in der Tranenflussigkeit. Dtsch Ges Wesen.

[5] Mollison PL, Engelfriet CP, Contreras M (1987). Blood Transf in clinical Medicine.

[6] Badakere SS, Bhatia HM (1971). ABO agglutinins in the saliva of Indians from Bombay. Proc Indian Soc Haematol Blood Transfusion.

[7] Tomasi T, Kunkel HG (1964). Isolation of 7s and 19s gamma globulins. meth. Med. Res 10. p80.

[8] Marsh WL (1972). Scoring of hemagglutination reactions. Transfusion.

[9] Schlesinger D, Osinska M (1964). The influence of pregnancy on salivary secretion of group isoantibodies. Arch Immunol Ther Exp (Warsz).

[10] Hanson LA, Söderström T (1981). Human milk: Defense against infection. Prog Clin Biol Res.

